# Assessment of the Hydrolysis of Pydiflumetofen and Its Degradation Characteristics in Agricultural Soils

**DOI:** 10.3390/molecules28114282

**Published:** 2023-05-23

**Authors:** Xianluo Shi, Guai Xie, Wei Zhang, Ailin Yu

**Affiliations:** 1 Forest Protection Science and Technology Innovation Team, Jiangxi Academy of Forestry, Economic and Technological Development Area, 1629 West Fenglin Road, Nanchang 330013, China; shixianluo66@163.com (X.S.); 18379995628@163.com (G.X.); 2 Jiangxi Water Resources Institute, Economic and Technological Development Zone, 99 Beishan Road, Nanchang 330013, China; 13197911690@163.com

**Keywords:** pydiflumetofen, hydrolysis, soil, degradation half-life

## Abstract

Pydiflumetofen is a potent fungicide that effectively inhibits pathogenic fungal growth by regulating succinate dehydrogenase activity. It effectively prevents and treats various fungal diseases, including leaf spot, powdery mildew, grey mold, bakanae, scab, and sheath blight. Pydiflumetofen’s hydrolytic and degradation properties were investigated indoors in four distinct soil types (phaeozems, lixisols, ferrosols, and plinthosols) to assess its risks in aquatic and soil environments. The effect of soil physicochemical properties and external environmental conditions on its degradation was also explored. Hydrolysis experiments found that pydiflumetofen’s hydrolysis rate decreased with increasing concentration, regardless of the initial concentration. Furthermore, an increasing temperature significantly enhances the hydrolysis rate, with neutral conditions having higher degradation rates than acidic and alkaline conditions. Pydiflumetofen showed a degradation half-life of 10.79–24.82 days and a degradation rate of 0.0276–0.0642 in different soils. Phaeozems soils had the fastest degradation, while ferrosols soils had the slowest. Sterilization significantly reduced its soil degradation rate and extended its half-life, which confirmed that microorganisms were the primary cause. Therefore, when using pydiflumetofen in agricultural production activities, the characteristics of water bodies, soil, and environmental factors must be considered, while minimizing the emissions and environmental impact.

## 1. Introduction

Agricultural chemicals or agrochemicals, an important product supporting agricultural production, are one or more chemical products for the prevention and control of plant diseases, pests, and weeds in agricultural, forestry, and animal husbandry activities, along with the regulation of plant growth [1,2]. After application, agricultural chemicals can end up in the water and soil through various channels and their hydrolysis and soil degradation are their important environmental properties [3,4]. Therefore, studying the hydrolytic and soil degradation behavior of agricultural chemicals is important for the scientific assessment of their use and safety. The hydrolysis process of agricultural chemicals belongs to the class of chemical reactions that involve interactions between the functional groups of the pesticide itself and those in the water, along with their microbial degradation, photolysis, and other behaviors in the water [5]. As early as 1993, it was discovered that the hydrolysis process of agricultural chemicals includes both single-molecule and two-molecule nucleophilic substitutions, with two types of hydrolytic reactions [6,7]. One involves agricultural chemicals having numerous easily hydrolyzable groups, such as lipids, pyridines, piperidines, pyrimidines, amides, and ureas, which undergo either direct or indirect hydrolysis in water to generate new metabolites. The other type contains numerous nucleophilic groups in natural water bodies, (OH^−^, Cl^−^, F^−^, SO_4_^2−^, NO_3_^−^, CO_3_^2−^, etc.), which bind pesticide groups (ester bonds, alkyl ester bonds, phosphate ester bonds, amide bonds, halogens, etc.), and undergo rapid hydrolysis to degrade pesticides [8,9,10,11]. Since most are sprayed directly onto the soil surface, the soil is the final destination of agricultural chemicals, thereby making it crucial to study their degradation in the soil [12]. After agricultural chemicals enter the soil, they undergo different environmental processing, including adsorption, desorption, volatilization, migration, leaching, hydrolysis, photolysis, and biodegradation, with soil degradation being the main cause of agricultural chemical reduction [13]. The degradation of agricultural chemicals in soil is influenced by various factors such as soil characteristics (pH, clay, organic carbon, organic matter, etc.), temperature, moisture content, microbial content, and light intensity [14,15,16].

**Figure 1:**
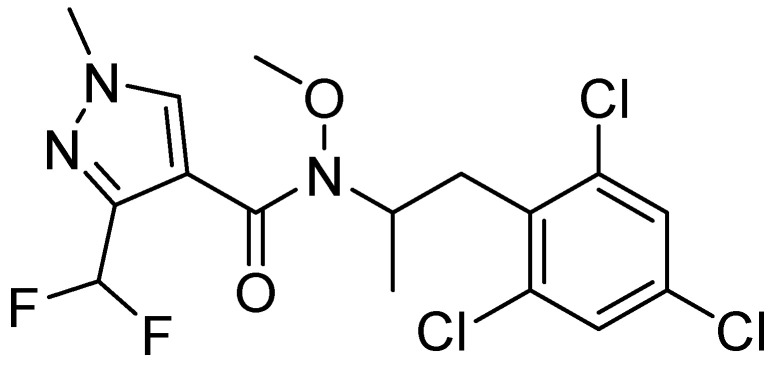
Chemical structural formula of pydiflumetofen.

Pydiflumetofen is a new generation succinate dehydrogenase inhibitor (SDHI) fungicide developed by Syngenta Crop Protection Co., Ltd. in Basel, Switzerland [17]. It inhibits the mitochondrial function by interfering with the tricarboxylic acid (TCA) cycle on the respiratory electron transfer chain complex II, which consequently hampers energy production, inhibits pathogen growth, and ultimately leads to its death (see chemical structural formula in Figure 1) [18,19]. This is an efficient and broad-spectrum agent, suitable for controlling various diseases in various crops, with good effects on wheat scab, soybean leaf spot, potato early blight, etc. [20]. Currently, global research on pydiflumetofen has focused on its efficacy, prevention and control, toxicology, and residual analysis methods [21,22]. Bian et al. studied how pydiflumetofen controlled rice sheath blight, while Liu et al. developed a QuEChERS ultra-high performance liquid chromatography–tandem mass spectrometry method to detect pydiflumetofen residues in various fruits and vegetables such as tomatoes, cucumbers, apples, etc. [23,24]. Furthermore, Sheng et al. found that pydiflumetofen had a low residue in watermelons and a high adsorption capacity in the soil [25]. Bian et al. established a pydiflumetofen analysis method exploring its environmental behavior in a rice paddy ecosystem [26,27].

This paper will not only investigate how pydiflumetofen is hydrolyzed in water but also its degradation behavior in four different agricultural soils. Here, not only were the hydrolysis patterns at different initial concentrations, temperatures, and pH values explored, but also the impact of soil microorganisms, organic matter, pH, temperature, humidity, and the initial amounts of addition on the degradation of pydiflumetofen in soil were evaluated. Therefore, this study provides a reference for an environmental safety assessment and risk assessment of pydiflumetofen after its agricultural application.

## 2. Results and Discussion

### 2.1. Precision Results, LOD, and LOQ

At different levels of addition, the average recovery rate of pydiflumetofen in the soil was 90.16% to 101.11%, with a relative standard deviation of 1.62–6.75%. When the recovery is between 70~120% and RSD < 15%, it indicates that the analytical residue method has a good accuracy and precision [28]. LOD is defined as the concentration of pydiflumetofen when the signal-to-noise ratio exceeds 3 (S/N = 3) in the matrix extraction, while LOQ is defined as the concentration of pydiflumetofen when the signal-to-noise ratio exceeds 3 (S/N = 10) in the matrix extraction [27,29]. The detection limit (LOD) and quantification limit (LOQ) were 0.0023–0.0033 mg/kg and 0.0082–0.0115 mg/kg, respectively (Table 1). This indicated that the analysis method meets the requirements of the “Guidelines for Pesticide Residues Experiment (NY/788-2018)” [28].

### 2.2. Hydrolysis Characteristics of Pydiflumetofen

#### 2.2.1. Hydrolysis at Different Initial Concentrations

**Figure 2:**
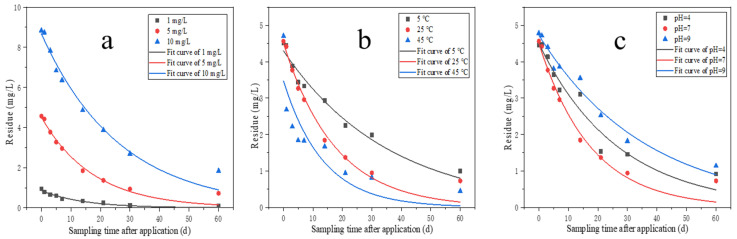
Hydrolysis kinetics curves of pydiflumetofen under different doses (**a**), temperature (**b**) and pH (**c**).

The hydrolysis kinetics curves and hydrolysis rate curves for pydiflumetofen at different initial concentrations are shown in Figure 2, while the hydrolysis kinetics model parameters are shown in Table 2. The results show that the pydiflumetofen hydrolysis rate decreases slightly with an increasing concentration. Pydiflumetofen has a half-life of 9.9–18.3 days at 1–10 mg/L with a hydrolysis rate constant of 0.0379–0.0702. According to the “Experimental Guidelines for Environmental Safety Assessment of Chemical Pesticides—Part 2: Hydrolysis Test”, it can be concluded that pydiflumetofen belongs to the category of easily degradable pesticides at different doses [30].

#### 2.2.2. Hydrolysis at Different Temperatures

The hydrolysis kinetics curves and hydrolysis rate curves for pydiflumetofen at different temperatures are shown in Figure 2, with the hydrolysis kinetics model parameters for different temperatures shown in Table 2. The pydiflumetofen hydrolysis rate increases with temperature. As can be seen from the fitting results, the first-order kinetic equation shows a better fit with the hydrolysis law of pydiflumetofen at different temperatures. The hydrolysis half-lives at 5 °C, 25 °C, and 45 °C was 24.8 days, 12.1 days, and 9.3 days, respectively. It can be seen that at the same pH value, the higher the temperature, the faster the pydiflumetofen degradation. For example, at pH 7, the hydrolysis rate constant at 45 °C was 1.30 times that at 25 °C, which in turn was ~2 times that at 5 °C.

#### 2.2.3. Hydrolysis at Various pH Values

The hydrolysis kinetics curves and hydrolysis rate curves for pydiflumetofen in different pH solutions are shown in Table 2 and Figure 2. Considering the three pH values, the pydiflumetofen degradation rate under neutral conditions was much higher than that under acidic and alkaline conditions, with the hydrolysis speed being in the following order: neutral > alkaline > acidic. For pH values of 4, 7, and 9 in the buffer solution, the pydiflumetofen degradation rates after 60 days of hydrolysis were 79%, 84%, and 76%, respectively. The first-order kinetic equation can be used to better fit the pydiflumetofen hydrolysis process under these three conditions (R^2^ > 0.94). Based on the hydrolysis rate constants, it was possible to calculate the hydrolysis half-lives for each treatment. These were 18.6, 12.1, and 25.0 days for the treatments with pH values of 4, 7, and 9, respectively. The hydrolysis process of pesticides generally mainly involves various chemical reactions between pesticides and water bodies. In hydrolysis, nucleophilic groups such as OH is substituted for electrophilic groups such as Cl, S, P [31]. Pydiflumetofen is hydrolyzed most rapidly under neutral conditions, primarily because the nucleophilic groups of pydiflumetofen are vulnerable to attack under neutral conditions, which cause ring opening reactions and molecular cleavage [32]. The hydrolysis of pesticides depends not only on the pH values but, more importantly, on factors such as the pesticide’s physicochemical properties, temperature, etc. [33,34].

### 2.3. Degradation Characteristics of Pydiflumetofen in Soils

#### 2.3.1. Effects of Different Soil Types on Pydiflumetofen Degradation

After a period of cultivation and sampling at different time intervals, the results show that with non-sterilized soil, the amount of pydiflumetofen added to the four types of soil gradually decreased with an increasing cultivation time. After data processing, it was found that the degradation follows first-order kinetics. Pydiflumetofen was calculated to have a half-life of 10.79 days, 20.43 days, 25.14 days, and 24.82 days in the four unsterilized soils. The corresponding degradation rate constants were 0.0642, 0.0339, 0.0276, and 0.0279, which indicated that the phaeozems soil had the fastest degradation, followed by lixisols soil, and ferrosols soil had the slowest. In contrast, the half-lives of pydiflumetofen in the sterilized soils were significantly prolonged (Table 3), with values of 26.3, 44.6, 55.4, and 52.5 days, and degradation rate constants of 0.0264, 0.0155, 0.0125, and 0.0132, respectively. Therefore, it can be seen that biodegradation plays a major role in the pydiflumetofen degradation process in soil [35].

From Table 3, it can be seen that the pydiflumetofen had a faster degradation rate in the phaeozems soil, while being slower in the other three types of sterilized soil, with the difference being negligible. However, the chemical degradation rate of pydiflumetofen was comparable to those of the other three soils due to the significant difference in OM contents between the membrane soil and the other three soils (all of which had similar organic matter content). Therefore, it can be inferred that soil OM also affected the pydiflumetofen degradation rate.

Furthermore, it can also be seen from the results in Table 4 that the degradation half-life of this pydiflumetofen showed the highest correlation with the soil OM content, followed by a high correlation with the pH value and the CEC, and finally a low correlation with the soil clay content. Presumably, both CEC and pH affected the microbial activity involved in degradation by affecting the soil environment. If the soil has a high CEC, this reflects its good manure retention ability and long-lasting nutrient supply. Moreover, if the pH is moderate, it is easier to form a good soil structure, with the availability of nitrogen, phosphorus, potassium, and other elements also being higher than in the acidic soils [12,16]. Therefore, these improved environmental conditions favor the growth of microorganisms and the degradation of pesticides.

#### 2.3.2. Effects of Different Moisture Content on Pydiflumetofen Degradation

As seen from Table 5, there are some differences in the pydiflumetofen degradation rates for different water contents of the ferrosol soil. Although the half-lives tended to shorten with the increasing water content, this tendency was not significant. The difference in half-life between treatments with 60% and 80% water contents was highly significant, while that between treatments with 40% and 60% water contents was not significant. Finally, the degradation rate in waterlogged conditions was 0.0429, which was significantly higher than the other treatments. Therefore, the pydiflumetofen degradation rate was found to increase with an increasing soil moisture content. While water promoted microbial growth, it also reduced soil adsorption to these pesticides, thereby increasing their bioavailability. Since this experiment created an anaerobic microenvironment under waterlogged conditions, it enhanced the anaerobic microbial activity [36]. Therefore, different soil moisture contents and permeabilities not only affect the soil reduction potential, but also the pydiflumetofen degradation rate.

#### 2.3.3. Effects of Different Temperatures on Pydiflumetofen Degradation

From Table 6, it can be seen that as the temperature increased, the pydiflumetofen degradation rate in ferrosol soil also increased, thereby indicating a positive correlation between its degradation rate in soil and the temperature. At temperatures ranging from 25 °C to 45 °C, the pydiflumetofen degradation rate significantly increased with the increase in temperature. Furthermore, the half-life reduced from 25.1 days to 18.2 days, while the degradation rate constant increased from 0.0276 to 0.0338. Temperature usually greatly affects the soil microbial activity, with the suitable temperature being 25–35 °C [37,38]. During pydiflumetofen degradation, while an increase in temperature promotes hydrolysis, it also enhances microbial activity and accelerates degradation.

#### 2.3.4. Effects of Different Initial Concentrations on Pydiflumetofen Degradation

From Table 7, it can be seen that besides the effects of water content, temperature, and humidity, the addition of pydiflumetofen concentration significantly affected the pydiflumetofen degradation in the ferrosol soil under the same cultivation conditions. The pydiflumetofen degradation rate significantly decreased with the increasing concentration. When the added concentration increased from 1 mg/L to 5 mg/L, the degradation half-life increased from 15.6 to 25.1 days, whereas the degradation rate constant decreased from 0.0444 to 0.0276. Furthermore, when the concentration increased from 5 mg/L to 10 mg/L, the degradation half-life increased from 25.1 to 29.5 days and the degradation rate constant decreased from 0.0276 to 0.0235. Therefore, it can be concluded that under other invariant conditions, the higher the initial concentration, the slower the degradation rate and the longer the half-life of pydiflumetofen in soil. High doses of pydiflumetofen may inhibit the activity and diversity of microorganisms in the soil. Studies have shown that the degradation rates of pesticides such as Dimethyl Disulfide and Allyl Isothiocyanate are significantly higher in low-dose soils than in high-dose soils, primarily because higher doses of pesticides affect the insecticide-degrading bacteria in the soil [39,40].

## 3. Materials and Methods

### 3.1. Chemicals and Soils

The following chemicals were used: (1) pydiflumetofen standard (≥98%, Syngenta Crop Protection Co., Ltd., Basel, Switzerland); (2) 88% formic acid (HPLC grade, Tianjin Kemio Chemical Reagent Co., Ltd., Tianjin, China); (3) sodium chloride (NaCl) and anhydrous magnesium sulfate (MgSO_4_) (analytical grade, Shanghai McLean Co., Ltd., Shanghai, China); acetonitrile (LC-MS grade, Shanghai Ampu Experimental Technology Co., Ltd., Shanghai, China); and octadecylsilane (C18, 50 μm) (Beijing Agela Technologies Co., Ltd., Beijing, China).

The test soils (Harbin phaeozems soil, Jining lixisols soil, Yichun ferrosols soil, and Haikou plinthosols soil) were collected from the 0–20 cm surface soil in September 2021. After removing impurities such as plant roots and crushed stones, they were naturally air-dried and ground through a 2.0 mm sieve and stored in polyethylene plastic bags for future use. The soil texture was determined using the hydrometer method, while the pH value was measured in the soil suspension (soil:water = 1:2.5, *w*/*v*) using the pH meter. The soil cation exchange capacity (CEC) was determined using the ammonium ion exchange method. The soil organic matter (OM) was measured by the dichromate digestion method. The basic physical and chemical properties of each soil are listed in Table 8, with no pesticide residues being detected in the four soils tested before the experiment.

### 3.2. Design of Hydrolysis Experiment for Pydiflumetofen

#### 3.2.1. Hydrolysis at Different Initial Concentrations

A water solution of pydiflumetofen with initial mass concentrations of 1, 5, and 10 mg/L and a final pH of 7 was prepared for the experiments. The solution was mixed well by stirring, then divided into brown glass bottles, and finally incubated separately in a constant temperature incubator at 25 °C in the dark for 60 days. Samples were taken at 0, 1, 3, 5, 7, 14, 21, 30, and 60 days post-culture to detect the residual amounts of pydiflumetofen in solution.

#### 3.2.2. Hydrolysis at Different Temperatures

The initial concentration for the preparatory experiments was 5 mg/L and the final pH value was 7. The aqueous solution of pydiflumetofen was mixed well by stirring. It was then divided into different brown glass bottles and incubated in constant temperature incubators at 5 °C, 25 °C, and 45 °C for 60 days in a dark environment. Samples were taken at 0, 1, 3, 5, 7, 14, 21, 30 and 60 days post-culture to detect the residual amounts of pydiflumetofen in the solution.

#### 3.2.3. Hydrolysis at Various pH Values

The pydiflumetofen working solution was added to the different buffer solutions with pH values of 5, 7, 9, and 11, respectively, such that the initial pydiflumetofen concentration was 5 mg/L. They were stirred to mix them well, then packed separately in brown glass bottles, and finally incubated in a constant temperature incubator at 25 °C in the dark for 60 days. Samples were taken at 0, 1, 3, 5, 7, 14, 21, 30, and 60 days post-culture to detect the residual amounts of pydiflumetofen in solution.

### 3.3. Design of Soil Degradation Experiments for Pydiflumetofen

#### 3.3.1. Effects of Different Soil Types on Pydiflumetofen Degradation

The four soils tested were divided into the sterilized and unsterilized treatment groups. First, 5.00 g of air-dried and screened soil were weighed and placed in a 50 mL polyethylene centrifuge tube. Then, 5 mg/kg pydiflumetofen was set and the relative soil moisture content was adjusted to 60%. These were then incubated in a dark incubator at 25 °C. Samples were taken at 0, 1, 3, 5, 7, 14, 21, 30, and 60 days after dosing to determine their residuals. Finally, an appropriate amount of water was added to the soil sample every 6–7 days to maintain the constant soil moisture. This was repeated three times for each treatment group.

The soil samples were sterilized intermittently at 121 °C thrice, each time for 20 min, in a high-temperature high-pressure sterilization pot. The test soil was weighed into a 50 mL volumetric flask, and an appropriate amount of ultrapure water was added to make a 60% relative water content of the soil. The bottle was sealed with a cotton plug and then incubated in a constant temperature incubator in darkness for 14 days.

#### 3.3.2. Effects of Different Moisture Contents on Pydiflumetofen Degradation

Pydiflumetofen concentration was also set at 5 mg/kg and the relative soil moisture content was adjusted to 40%, 60%, 80%, and waterlogged gradients. This was repeated thrice for each treatment group.

#### 3.3.3. Effects of Different Temperatures on Pydiflumetofen Degradation

Pydiflumetofen was added at a concentration of 5 mg/kg and the relative soil moisture content of 60% was maintained. It was then cultured in a dark incubator at 5 °C, 25 °C, and 45 °C. This was repeated thrice for each treatment group.

#### 3.3.4. Effects of Different Initial Concentrations on Pydiflumetofen Degradation

Three gradient concentrations of pydiflumetofen were set: 1, 5, and 10 mg/kg. The relative moisture content of the soil was adjusted to 60% and cultivated in a dark incubator at 25 °C. This was repeated thrice for each treatment group.

### 3.4. Sample Extraction, Purification, and Determination

#### 3.4.1. Sample Extraction

First, 5 mL of water sample was added to a 50 mL centrifuge tube, followed by adding 10 mL of 0.1% formic acid acetonitrile to it and vortexing for 2 min. Then, 1 g of NaCl and 2 g of MgSO_4_ was added, then vortexed for 1 min, and finally centrifuged for 5 min at 9000× *g* rpm and then purified.

For the soil sample, first, 5 g of it was added to a 50 mL centrifuge tube along with 5 mL of ultrapure water and 10 mL of 0.1% formic acid–acetonitrile solution. These were then vortexed at 3000× *g* rpm for 2 min. Finally, 1 g NaCl and 2 g MgSO_4_ was added to the mixture, vortexed for 1 min, then centrifuged for 5 min at 9000× *g* rpm and then purified.

#### 3.4.2. Sample Purification

Here, 1.5 mL of the upper clear supernatant was added to a 2.5 mL centrifuge tube (containing 150 mg MgSO_4_ + 50 mg PSA). These were vortexed for 1 min, centrifuged at 6000× *g* rpm for 5 min, and passed through a 0.22 μm sterile filter for it to be tested by HPLC-MS.

#### 3.4.3. Sample Determination

The following equipment was used: Agilent 1260 HPLC with auto-sampler (USA), Zorbax Eclipse XDB-C18 chromatographic column (4.6 mm × 150 mm, 5 µm), Agilent 6120 mass spectrometer (USA), 0.22 µm filter membranes, Metler ME204 analytical balance (0.1 mg, Beijing, China), Eppendorf 5804R high-speed freezing centrifuge (Ebende, Germany), XH-C vortex mixer (Kunshan, China), FW80 high-speed Universal Crusher (Beijing, China), and SB-5200DT ultrasonic cleaner (Ningbo, China).

**Figure 3:**
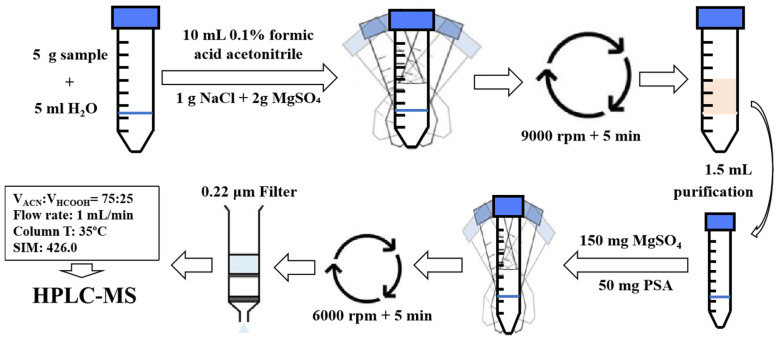
A schematic summary of the recommended protocol for the extraction and purification of pydiflumetofen.

The following main parameters were set as follows: 10 μL sample injection volume; the mobile phase was acetonitrile: 0.1% formic acid water (75:25, *v*/*v*); column temperatures were 35 ºC, and a flow rate 1 mL/min. At the same time, the ESI ion source was ESI+, with a dwell time of 590 ms; relative residence = 100.0%; collision induced dissociation voltage = 105 V; gain = 10.00; mass-to-charge ratio (*m*/*z*) under SIM mode = 426.0; liquid gas flow rate = 12.0 L/min. The pressure of the accreted gas was 35 psig. Under these detection conditions, the retention time of each sample was ~4.336 min. The specific process was shown in Figure 3.

#### 3.4.4. Preparation of Standard and Matrix Solutions

A standard sample of 0.0100 g pydiflumetofen was accurately weighed and dissolved in a 100 mL volumetric flask with a small amount of acetonitrile. This was followed by dissolving it in an ultrasonic water bath for 10 min. After cooling, the volume was adjusted to the scale line with acetonitrile and then uniformly shaken to prepare a standard solution of 100 mg/L. A series of standard solutions of 0.01, 0.05, 0.1, 0.5, 1, and 5 mg/L were subsequently obtained by serial dilution with acetonitrile. Blank water and soil samples were weighed to obtain a pre-processed blank matrix acetonitrile solution, while the standard solution was diluted and a series of matrix standard solutions of 0.01, 0.05, 0.1, 0.5, 1, and 5 mg/L were prepared.

#### 3.4.5. Precision and Accuracy

Recovery experiments were performed for each blank sample with concentrations set to 0.01, 0.05, and 0.1 mg/kg. Five parallel experiments were performed at different levels of addition. Then, after subjecting the sample through the previous preprocessing steps, its recovery rate was determined. The accuracy and precision of the method were evaluated in terms of recovery rate and relative standard deviation.

### 3.5. Data Analysis and Statistics

To facilitate the pydiflumetofen degradation reactions in water and soil, a first-order kinetic model was used to describe the degradation process and the dissipative kinetic parameters were obtained by nonlinear fitting using the OriginPro 2018 software (OriginLab, Northampton, MA, USA). The formula is as follows:

C_t_ = C_0_× exp^−kt^

t_0.5_ = ln_2_/k

where “C_t_” represents the pesticide residue at time t (mg/kg); “C_0_” is the initial residual amount of pesticide after application (mg/kg); K” and “t” represent the degradation coefficient and the time after application (d), respectively; and “t_0.5_” represents the degradation half-life.

## 4. Conclusions

Hydrolysis tests have shown that the half-life of pydiflumetofen hydrolysis increases with the increasing initial concentration, while its hydrolysis rate increased with the increasing temperature. The hydrolysis of pydiflumetofen was the fastest under neutral conditions, while being relatively stable under alkaline conditions. Through soil degradation experiments, it has been shown that the pydiflumetofen degradation in soil was not only influenced by microorganisms, but also by the physicochemical properties of the soil, including the OM content and CEC. Additionally, the pydiflumetofen degradation rate increased with the increasing water content. Temperature is one of the important factors determining the pydiflumetofen degradation. The increase in temperature not only promotes its hydrolysis, but also enhanced microbial activity, which accelerated its degradation. Consistent with its hydrolysis pattern, the pydiflumetofen degradation rate also slowed down as the initial concentration increased. Therefore, in agricultural production, high doses must be avoided as much as possible to reduce not only the residual time and amount of pydiflumetofen accumulating in the plants and soil, but also environmental pollution.

## Figures and Tables

**Table 1:** Linear equations, coefficient of determination, detection limit, and quantification limit of pydiflumetofen in different soil matrixes.

Matrix	Regression Equation	R^2^	LOD (mg/kg)	LOQ (mg/kg)
Acetonitrile	y = 438476x − 9154.5	0.9999	-	-
Water	y = 395146.95 x + 4537.86	0.9999	0.0019	0.0063
phaeozems	y = 395091x + 6260	0.9998	0.0033	0.0115
lixisols	y = 421776x − 8302.6	0.9998	0.0025	0.0089
ferrosols	y = 405017x + 13869	0.9997	0.0031	0.0108
plinthosols	y = 427307x − 4205.9	0.9998	0.0023	0.0082

**Table 2:** Hydrolysis curve equations and half-lives of pydiflumetofen under different conditions.

Classification	Treatment Group	Rate Constant, k	R^2^	Hydrolysis Kinetic Equation	Half-Life, t_1/2_,d
Different temperature	5	0.0280	0.9663	C_t_ = 4.3014e^−0.0280t^	24.8
	25	0.0571	0.9715	C_t_ = 4.5121e^−0.0571t^	12.1
	45	0.0742	0.7251	C_t_ = 3.4726e^−0.0742t^	9.3
Different pH	pH = 4	0.0373	0.9424	C_t_ = 4.5000e^−0.0372t^	18.6
	pH = 7	0.0571	0.9715	C_t_ = 4.5121e^−0.0571t^	12.1
	pH = 9	0.0277	0.9689	C_t_ = 4.7333e^−0.0277t^	25.0
Different doses	1	0.0702	0.9593	C_t_ = 0.8605e^−0.0702t^	9.9
	5	0.0571	0.9715	C_t_ = 4.5121e^−0.0571t^	12.1
	10	0.0379	0.9712	C_t_ = 8.6758e^−0.0379t^	18.3

**Table 3:** Equations for the degradation curves and half-lives of pydiflumetofen in four soils.

Soil Type	Treatment	Degradation Kinetic Equation	R^2^	Rate Constant, k	Half-Life, t_1/2_,d
Phaeozems	Sterilized	ct = 3.6594e^−0.0264t^	0.9856	0.0264	26.27
	Non-sterilized	ct = 4.3570e^−0.0642t^	0.9804	0.0642	10.79
Lixisols	Sterilized	ct = 4.1437e^−0.0155t^	0.9158	0.0155	44.6
	Non-sterilized	ct = 4.3479e^−0.0339t^	0.8952	0.0339	20.43
Ferrosols	Sterilized	ct = 3.8283e^−0.0125t^	0.9409	0.0125	55.41
	Non-sterilized	ct = 4.2430e^−0.0560t^	0.9279	0.0276	25.14
Plinthosols	Sterilized	ct = 3.8151e^−0.0132t^	0.9226	0.0132	52.51
	Non-sterilized	ct = 4.0279e^−0.0279t^	0.9284	0.0279	24.82

**Table 4:** Correlation between degradation half-life and soil properties.

Soil Properties	Correlative Equation	R^2^
OM (%)	y = −1.3381x + 26.18	0.9784
pH	y = −5.8668x + 54.514	0.8723
CEC (cmol/kg)	y = −0.6782x + 31.492	0.8826
Clay (%)	y = −0.283x + 31.364	0.4777

**Table 5:** Equations for the degradation curves and half-lives of pydiflumetofen in different moisture contents.

Treatment	Degradation Kinetic Equation	R^2^	Rate Constant, k	Half-Life,t_1/2_,d
40%	ct = 4.0739e^−0.0263t^	0.9786	0.0263	26.35
60%	ct = 4.2430e^−0.0276t^	0.9279	0.0276	25.14
80%	ct = 3.8283e^−0.0379t^	0.9408	0.0379	18.26
waterlogged	ct = 2.5754e^−0.0429t^	0.9221	0.0429	16.15

**Table 6:** Equations for the degradation curves and half-lives of pydiflumetofen in different temperatures.

Treatment	Degradation Kinetic Equation	R^2^	Rate Constant, k	Half-Life, t_1/2_,d
5 °C	ct = 3.9222e^−0.0195t^	0.8457	0.0195	26.35
25 °C	ct = 4.2430e^−0.0276t^	0.9278	0.0276	25.14
45 °C	ct = 3.7640e^−0.0338t^	0.9241	0.0338	18.26

**Table 7:** Equations for the degradation curves and half-lives of pydiflumetofen in different initial concentrations.

Treatment	Degradation Kinetic Equation	R^2^	Rate Constant, k	Half-Life,t_1/2_,d
1 mg/L	ct = 0.9214e^−0.0502t^	0.8457	0.0444	15.6
5 mg/L	ct = 4.2430e^−0.0276t^	0.9278	0.0276	25.14
10 mg/L	ct = 6.2821e^−0.2797t^	0.9241	0.0235	29.48

**Table 8:** Sampling locations and physicochemical properties of the four different agricultural soils.

Soil Locations	Soil Category	Texture				pH	CEC (cmol/kg)	OM (%)
		Sand (%)	Silt (%)	Clay (%)	Texture Class			
Haerbin, Heilongjiang (41°36′ N, 127°53′ E)	phaeozems	13.18 ± 1.23 D	32.41 ± 2.43 B	54.41 ± 5.72 A	Clay loam	7.28 A	30.36 A	11.68 A
Jining, Shandong (36°40′ N, 117°02′ E)	lixisols	30.76 ± 3.42 B	26.99 ± 2.72 C	42.24 ± 4.03 B	Sandy loam	5.31 B	11.78 B	3.28 B
Yichun, Jiangxi (27°82′ N, 114°42′ E)	ferrosols	15.87 ± 1.59 C	40.30 ± 3.57 A	43.83 ± 3.72 B	Silt loam	5.21 B	12.89 B	1.52 C
Haikou, Hainan (19°32′ N, 110°10′ E)	plinthosols	51.05 + 5.32 A	32.97 ± 4.02 B	15.98 ± 1.61 C	Silt loam	5.19 C	11.00 B	1.21 C

CEC, cation exchange capacity; OC, organic carbon; OM, organic matter. A–D represents a significant difference.

## Data Availability

Not applicable.

## Sample Availability

Samples of the compounds are not available from the authors.
